# Trauma-related symptoms in adolescents: the differential roles of sexual abuse and mentalizing

**DOI:** 10.3389/fpsyg.2024.1364001

**Published:** 2024-07-03

**Authors:** Marissa Wais, Michaël Bégin, Carla Sharp, Karin Ensink

**Affiliations:** ^1^École de Psychologie, Université Laval, Québec, QC, Canada; ^2^Département de Psychologie, Université de Sherbrooke, Sherbrooke, QC, Canada; ^3^Department of Psychology, University of Houston, Houston, TX, United States

**Keywords:** mentalizing, childhood sexual abuse, posttraumatic stress, borderline personality features, sexual concerns

## Abstract

**Introduction:**

Major gaps remain in our knowledge regarding childhood sexual abuse (CSA) related symptoms in adolescent psychiatric inpatients, as well as potential resilience factors like mentalizing. CSA is a risk factor for the early emergence of borderline personality features, posttraumatic stress, and sexual concerns. Mentalizing, which involves the capacity to understand our reactions and that of others in psychological terms, is a resilience factor for self and interpersonal functioning. The aim of this study was to address knowledge gaps by examining the contributions of CSA and mentalizing in a latent factor composed of borderline personality features, posttraumatic stress, and sexual concerns in a sample of adolescent psychiatric inpatients. We hypothesized that CSA and mentalizing would independently explain the variance in this latent factor.

**Method:**

Participants were 273 adolescents aged 12–17 recruited from an adolescent inpatient psychiatric clinic. They completed the Reflective Function Questionnaire for Youth (RFQ-Y), the Trauma Symptom Checklist for Children (TSCC), and the Borderline Personality Features Scale for Children (BPFS-C). CSA was assessed using the Child Attachment Interview (CAI), the Computerized Diagnostic Interview Schedule for Children (C-DISC), as well as the Childhood Trauma Questionnaire (CTQ).

**Results:**

27.5% of adolescent psychiatric inpatients reported CSA. CSA and mentalizing were independently associated with a latent factor consisting of posttraumatic stress, borderline personality features, and sexual concerns. CSA explained 5.0% and RF explained 16.7% of the variance of the latent factor. When we consider both the unique and the shared contribution of CSA and mentalizing, the model explained 23.0% of the variance of this factor.

**Discussion:**

CSA and mentalizing independently explained variance in a latent factor constituted of borderline personality features, posttraumatic stress, and sexual concerns. The direct effect of mentalizing was stronger and mentalizing explained comparatively more variance of trauma-related symptoms in adolescent psychiatric inpatients. The findings are consistent with the theory that mentalizing is an internal resilience factor in adolescent psychiatric inpatients. By implication, clinical interventions focused on promoting the development of mentalizing, such as Mentalization Based Treatment, may palliate mental health difficulties manifested by adolescent psychiatric inpatients including those associated with CSA.

## 1 Introduction

Sexual violence towards children and youth is a major public health concern contributing to a high disease burden, with 70–75% of survivors developing mental health problems (Trickett et al., 2011; Pérez-Fuentes et al., 2013). Furthermore, CSA accounts for 47% of childhood-onset psychiatric disorders and approximately 30% of adult-onset disorders (Green et al., 2010). Less is known regarding the association between CSA and psychiatric problems during adolescence, a critical development period for the emergence of psychopathology. Ford and Courtois (2021) have suggested that maltreatment, including CSA, is frequently associated with complex trauma symptoms, Posttraumatic Stress Disorder (PTSD), and Borderline Personality Disorder (BPD), where PTSD and BPD can be seen as progressively more severe trauma sequelae. Trauma can disrupt adolescent personality development (Ensink et al., 2024), putting CSA survivors at a significantly increased risk of developing BPD (Cutajar et al., 2010). Early emergence of BPD is consistently associated with CSA across studies, with 26.0% of adolescents and 62.4% of adult inpatients with BPD reporting CSA (Bryer et al., 1987; Herman et al., 1989; Zanarini et al., 1989, 1997; Ogata et al., 1990; Shearer et al., 1990; Westen et al., 1990; Salzman et al., 1993; Paris et al., 1994a,b; Silk et al., 1995; Laporte and Guttman, 2001; Merza et al., 2015; Reed et al., 2015; de Aquino Ferreira et al., 2018). In addition, CSA survivors are at elevated risk of developing trauma-related symptoms with 54% developing PTSD (Nooner et al., 2012). CSA is also a significant predictor of comorbid BPD and PTSD (Scheiderer et al., 2015).

In the context of inpatient treatment, a recent study of adolescent psychiatric inpatients found a 28.5% prevalence of CSA with 80% presenting with personality disorders and 30% with PTSD (Robin et al., 2023). Adolescent inpatients with CSA and BPD have more frequent hospitalisations, cumulative hospital stays five times longer than average, are more likely to manifest suicidality (Horesh et al., 2009; Kaplan et al., 2016), and are less likely to remit after 4 years (Biskin et al., 2011). In sum, this suggests that adolescents with BPD and CSA manifest more severe difficulties, or that CSA-related trauma is not optimally treated.

Given CSA’s sexual and interpersonal nature, it is associated with ranging harmful impacts on sexual development and romantic relationships, such that sexual concerns are considered a CSA-related trauma impact (Briere and Elliott, 2003; Gewirtz-Meydan and Lassri, 2023) and even a CSA-related complex trauma symptom (Abu-Raya and Gewirtz-Meydan, 2023). Sexual concerns are evident from childhood in the form of persistent sexualized behaviors, which are manifested by 50% of child CSA survivors (Ensink et al., 2018). During adolescence, CSA is known to be associated with an earlier onset of sexual relations, risky-sex, and revictimization (Fergusson et al., 1997). CSA-related sexual concerns may also hinder the development of stage salient sexual and romantic relationships during adolescence. Better knowledge is needed to improve care for adolescent CSA survivors.

Not all CSA survivors develop mental health problems. External protective factors include parents with high mentalizing capacities who respond sensitively to distress and attachment needs, which are activated by abuse, thus providing safety and security (Ensink et al., 2016, 2017a,b,c,d, 2020). Internal capacities, such as attachment and mentalizing, are also considered resilience factors in the context of trauma (Ensink et al., 2016, 2021; Borelli et al., 2018) because they promote interpersonal functioning and emotional regulation. However, significant knowledge voids exist regarding CSA, mentalizing, and trauma-related symptoms in adolescence.

### 1.1 Mentalizing

Mentalizing involves the capacity to understand our reactions and those of others in psychological terms (Fonagy et al., 2018). Mentalizing enables us to see ourselves from the outside, to imagine the impact of our emotional reactions and behaviors on others, and to predict how others might feel and behave in response. Through making interpersonal reactions comprehensible and predictable, mentalizing facilitates social connections, interpersonal relationships, and self-regulation (Fonagy and Luyten, 2009). Mentalizing has been operationalized as reflective functioning (RF) for research purposes. RF is mentalizing’s quantifiable counterpart and is used interchangeably with mentalizing (Fonagy and Target, 1997; Fonagy et al., 1998).

In the context of normal development, children from the ages of eight and onwards use psychological and mental state terms to describe themselves and their personalities, as well as think about their relationships with attachment figures in psychological terms. They are generally able to imagine the experience of others, consider the impact of their own behaviors on others, understand interpersonal interactions and relationships in mental state terms, and explain the reactions and behaviors of attachment figures and others in terms of motivations and intentions (Ensink et al., 2015a,b). Mentalizing rapidly increases in sophistication during adolescence, a developmental period in which mentalizing becomes more complex and adult-like (Poznyak et al., 2019). This is driven by significant functional and structural maturation of the “social brain,” a network of brain regions involved in social-cognitive processes (Choudhury et al., 2006; Blakemore, 2008; Crone and Dahl, 2012; Taylor et al., 2013). The rapidly expanding social worlds of adolescents provide rich opportunities for learning about others’ minds and mental states (Venta and Sharp, 2015; Poznyak et al., 2019).

#### 1.1.1 Mentalizing and its development in the context of CSA and maltreatment

Children learn mentalizing in the context of attachment relationships from parents who see them as psychological beings and interpret their behavior in terms of underlying psychological motivations (Fonagy and Target, 2006; Fonagy et al., 2007). In families where maltreatment occurs, parental mentalizing is generally low or distorted, with a focus on behavior rather than the psychological experiences of the child (Ensink et al., 2017a,b,c,d). The act of maltreatment is incompatible with imagining the child’s distress and psychological experience. Mentalizing impacts have been demonstrated empirically in the context of CSA, where Ensink et al. (2015a,b) showed that child mentalizing was predicted by parental mentalizing in a sample of child CSA survivors and a matched control group. Parents of child CSA survivors manifested significantly lower abilities to mentalize about their relationships with their children than parents of the non-abused control group. In addition, child CSA survivors aged 8–12 manifested significantly lower mentalizing regarding others compared with nonabused children. Furthermore, children who experienced intra-familial CSA manifested significantly lower mentalizing about themselves compared to those who experienced extra-familial CSA. When the abuser is an attachment figure, their malevolent intentions and the abuse itself may lead the child to defensively inhibit mentalizing in order to reduce anxiety and maintain their relationship with the attachment figure, on whom the child is dependent for survival (Fonagy, 2004; Allen, 2018).

To our knowledge, a clear relation between CSA and mentalizing has not been demonstrated in adolescents. Recent studies with adolescents have reported a relation between mentalizing and maltreatment (including CSA) more broadly. For example, a recent meta-analysis (Yang and Huang, 2024) reported consistent negative associations between mentalizing and maltreatment in adolescent psychiatric outpatients and inpatients with BPD across several studies (Taubner et al., 2016; Quek et al., 2017; Penner et al., 2019; Adler et al., 2021). Duval et al. (2018) found that adolescents manifested significantly more uncertainty regarding mental states if they reported either CSA or physical abuse. Similarly, adolescents and young adults who endorsed maltreatment manifested greater uncertainty regarding mental states (Doba et al., 2022). While these studies demonstrate that mentalizing and maltreatment are still very much linked in adolescence, we are not aware of any associations between mentalizing and CSA specifically.

#### 1.1.2 Mentalizing and trauma-related symptoms

Mentalizing is considered a transdiagnostic risk factor for psychopathology (Fonagy et al., 2016; Luyten et al., 2020). In the case of BPD in particular, mentalizing difficulties associated with insecure attachment and maltreatment are theorized to underlie the interpersonal difficulties and emotion dysregulation characteristic of BPD (Fonagy and Bateman, 2007; Chiesa and Fonagy, 2014). Adolescents with BPD have been shown to manifest more mentalizing difficulties, including over-interpretation of social cues (Sharp et al., 2011, 2013; Sharp and Vanwoerden, 2015), more uncertainty about others’ mental states or excessive certainty (Duval et al., 2018; Martin-Gagnon et al., 2023). Mentalizing was also shown to mediate the relationship between emotional abuse and borderline personality features in adolescents (Duval et al., 2018; Martin-Gagnon et al., 2023), further adding to the body of research demonstrating associations between mentalizing and BPD.

More recently, mentalizing difficulties have also been shown to be associated with posttraumatic stress (Huang et al., 2020; Doba et al., 2022; Ensink et al., 2023). In particular, mentalizing has been demonstrated to impact posttraumatic stress symptoms via emotion regulation in adolescents and young adults (Doba et al., 2022). In adult survivors of childhood maltreatment, mentalizing was found to be associated with posttraumatic stress and also mediated the relationship between attachment and posttraumatic stress symptoms (Ensink et al., 2023). Taken together, mentalizing may be an important resilience factor against the development of posttraumatic stress symptoms, including within the context of childhood maltreatment, in adolescence, and into adulthood.

To our knowledge, no previous studies have examined the relation between mentalizing and sexual concerns in the context of CSA in adolescents. However, we have previously shown that mentalizing and dissociation sequentially mediated the effects of CSA on sexualized behavior in children (Ensink et al., 2017a,b,c,d). Bigras et al. (2020) has theorized that CSA-related impacts on mentalizing and personality organization or identity may contribute to sexual concerns and difficulties with intimacy. More information is needed regarding the links between CSA, mentalizing, and sexual concerns in order to inform and improve clinical interventions.

### 1.2 The present study

To improve psychiatric care for adolescent psychiatric inpatients, a better understanding of CSA-related mental health difficulties and potential resilience factors, such as mentalizing, is needed. Mentalizing is of particular interest given that (1) it may be a resilience factor in the context of trauma, (2) underdeveloped mentalizing is a transdiagnostic risk factor for psychopathology, and (3) it is the focus of treatments such as Mentalization Based Treatment that have been shown effective for treating BPD. We focused on the relationship between CSA, mentalizing, borderline personality features, posttraumatic stress, and sexual concerns. The focus on borderline personality features and posttraumatic stress was motivated by findings showing that adolescent psychiatric inpatients who are CSA survivors frequently present with BPD and PTSD (Robin et al., 2023). We included sexual concerns because it is considered a CSA-related trauma symptom (Briere and Elliott, 2003; Gewirtz-Meydan and Lassri, 2023) and because of its particular relevance as a stage salient symptom.

To address current knowledge gaps, the aim of the present study was to clarify the relationships between CSA, mentalizing, and trauma-related symptoms, including posttraumatic stress, borderline personality features, and sexual concerns. A preliminary objective was to determine the prevalence of CSA in an adolescent psychiatry sample. Our main objective was to test a model where both CSA and mentalizing would have direct effects on a latent factor of trauma-related symptoms including posttraumatic stress, borderline personality features, and sexual concerns. Based on previous research showing associations between CSA and trauma-related symptoms, we hypothesized that CSA would have a direct positive effect and be associated with more trauma-related symptoms. In addition, we hypothesized that mentalizing would also have a negative direct effect on trauma-related symptoms, where better mentalizing would be associated with fewer trauma-related symptoms.

## 2 Methodology

### 2.1 Participants and procedure

The present study included a sample of 12–17 year-olds both with and without a history of CSA who were admitted to the adolescent unit of a private inpatient psychiatric hospital. Participants were recruited from a treatment program for adolescents with complex mental health problems who did not respond to previous interventions, had severe behavioral and emotional disorders, or comorbidity. Adolescents and their families were approached for consent upon admission to the hospital. Parental consent was obtained first, followed by adolescent assent. Exclusion criteria included diagnoses of schizophrenia, IQ < 70, and/or active psychosis to ensure participant comprehension of study materials. Ethics approval for the study was obtained by the appropriate institutional review boards.

The sample consisted of 273 adolescent inpatients (178 girls and 95 boys; mean age = 15.5, SD = 1.4). In terms of ethnicity, the majority of the participants were white (87.7%), with a smaller proportion identifying as Hispanic or Latino (4.8%), Asian (3.3%), Black or African-American (2.8%), and other/multiracial (5.5%). Assessments were administered by doctoral-level clinical psychology students, licensed clinicians, and trained clinical research assistants. The assessment process involved completing self-report questionnaires and structured clinical interviews during the adolescent’s stay.

### 2.2 Measures

#### 2.2.1 Mentalizing

##### 2.2.1.1 Reflective function questionnaire for youths (RFQ-Y)

The RFQ-Y is a 46-item self-report measure assessing reflective functioning in adolescents (Sharp et al., 2009). Items are scored on a six-point Likert scale ranging from ‘strongly disagree’ to ‘strongly agree,’ with two 23-item subscales, scale A and scale B, which use two different scoring approaches. In scale A, midpoint responses (i.e., agree somewhat or disagree somewhat) indicate higher mentalizing, while responses located near either endpoint (i.e., strongly agree or strongly disagree) indicate lower mentalizing. For example, a midpoint response in the statement “I always know what others are thinking,” would signify higher mentalizing due to having an appropriate amount of uncertainty about others’ minds. In scale B, responses located near one endpoint (i.e., strongly agree) indicate higher mentalizing, while responses located near the other endpoint (i.e., strongly disagree) indicate lower mentalizing. For example, strongly agreeing with “I like to think about the reasons behind my actions,” would correspond with higher mentalizing. Reverse coding was used for some items (e.g., “I get confused when people talk about their feelings.”). The sum of both scales was used to calculate the RFQ-Y total score. The RFQ-Y has demonstrated good criterion, convergent, and construct validity (Ha et al., 2013). In the present sample, internal consistency was acceptable with a Cronbach’s alpha of 0.78.

#### 2.2.2 CSA

We previously found using the same sample that CSA endorsement across multiple measures demonstrated only moderate agreement (Wais, 2022). Different operationalizations of CSA used across measures and different methodologies, such as a self-report questionnaire regarding trauma versus disclosure in the context of an interview, are thought to contribute to these differences. For this reason, we used information obtained from three evaluations to establish the presence of CSA. Participants who endorsed CSA on any of the three following measures were considered to have a history of CSA. As such, CSA history will be coded as a dichotomous variable, with a history of CSA = 1 and not having a history of CSA = 0.

##### 2.2.2.1 Child attachment interview (CAI)

The CAI is a 15-question assessment protocol designed to assess relationships with attachment figures and with oneself (Shmueli-Goetz et al., 2008). The CAI has been validated for use with adolescents (Venta et al., 2014) in both clinical and community samples (Shmueli-Goetz et al., 2008). Using the methodology of Bailey et al. (2007) and Jardin et al. (2017), the CAI was used uniquely to assess the presence of CSA. Specifically, an affirmative response to the question “Have you ever been touched sexually by someone when you did not want them to do it?” was considered indicative of CSA. If the child or adolescent answers “yes,” interviewers ask for more information regarding the abuse, but only if the adolescent is comfortable to do so. In this study, the CAI was used only to code the presence or absence of CSA. The CAI demonstrates concurrent validity in community, clinical, and CSA samples (Target et al., 2003; Shmueli-Goetz et al., 2008) and inter-rater reliability among expert and “naive” coders have been demonstrated to be acceptable (Shmueli-Goetz et al., 2008).

##### 2.2.2.2 Computerized diagnostic interview schedule for children (C-DISC)

The C-DISC is a structured computerized interview used to assess Axis 1 psychiatric disorders in children and adolescents aged 9–17 (Shaffer et al., 2000). The C-DISC is a well-established measure of Axis I psychopathology in youth and has been demonstrated to have good reliability and validity (Shaffer et al., 2000). Several questions are used to assess the respondent’s history of trauma. The item “Have you ever been very upset by someone forcing you to do something sexual that you really did not want to do?” was used to code for CSA.

##### 2.2.2.3 Childhood trauma questionnaire (CTQ)

The CTQ is a widely used 28-item self-report measure used to evaluate different types of childhood maltreatment, including CSA (Bernstein et al., 2003). Each item is rated on a Likert scale ranging from 0 (never true) to 5 (always true), with higher scores indicating increasingly severe maltreatment. For the sexual abuse subscale, a score of 6–7 indicates sexual abuse that is low to moderate, 8–12 indicates moderate to severe, and 13 or greater indicates severe to extreme. In this study, scores of 6 (low to moderate sexual abuse) and higher were considered indicative of CSA. In the current sample, Cronbach’s alpha for the Sexual Abuse subscale was α = 0.95.

#### 2.2.3 Trauma-related symptoms

##### 2.2.3.1 Trauma symptom checklist for children (TSCC)

The TSCC is a 54-item self-report measure that assesses posttraumatic symptomatology in children and adolescents (Briere, 1996). Trauma symptoms such as “Cannot stop thinking about something bad that happened to me,” are rated on a four-point frequency scale ranging from 0 (never) to 3 (almost all of the time). The TSCC comprises six clinical subscales: Posttraumatic Stress, Anxiety, Depression, Anger, Dissociation, and Sexual Concerns. Our variables of interest were the Posttraumatic Stress and Sexual Concerns subscales. The Posttraumatic Stress subscale evaluates common PTSD symptomatology, while the Sexual Concerns subscale evaluates distressing trauma-related sexual thoughts and behaviors. Strong internal consistency was observed in both the Posttraumatic Stress subscale (α = 0.89) and the Sexual Concerns subscale (α = 0.82).

##### 2.2.3.2 Borderline personality features scale for children (BPFS-C)

The BPFS-C is a 24-item self-report questionnaire for assessing borderline personality features in youth of at least 9 years old (Crick et al., 2005). This measure was adapted from the BPD scale of the Personality Assessment Inventory (PAI) (Morey, 1991) and uses the same four subscales: Affective Instability, Identity Problems, Negative Relationships, and Self-Harm. There are 6 items per subscale and each item is rated on a 5-point Likert scale ranging from 1 (“not true at all”) to 5 (“always true”). The total score is the sum of all items, with higher scores indicating a greater amount of borderline personality features. The BPFS has been demonstrated to have strong criterion and concurrent validity (Chang et al., 2011; Haltigan and Vaillancourt, 2016), and had strong internal consistency in the present sample (α = 0.90).

### 2.3 Data analysis

Bivariate correlations were used to examine the associations between RF and trauma-related symptoms including borderline personality features, posttraumatic stress, and sexual concerns. Point-biserial correlations were used to examine the associations between CSA, RF, and trauma-related symptoms. In order to examine the relative contribution of CSA and RF to different outcomes, structural equations modeling was conducted. The measurement model included a latent variable (trauma-related symptoms) predicting posttraumatic stress, sexual concerns, and borderline personality features. The structural model aimed at comparing the relative contribution of CSA and RF to trauma-related symptoms using an ML estimator. Different fit indices were used to test the adequacy of the model: the Comparative Fit Index (CFI), the Tucker-Lewis Index (TLI), the root mean square error of approximation (RMSEA) and the standardized root mean square residuals (SRMR), and the ratio of chi-square to degrees of freedom (*χ2/df*). Guidelines suggest that values above 0.95 for the CFI and TLI (Hoyle, 1995) and values below 0.05 for the RMSEA and SRMR, as well as a non-significant chi-square test, indicate an excellent fit (Browne and Cudeck, 1992; Ullman, 2004). There was no systematic missing data pattern, and consequently missing data was handled using the built-in full information maximum likelihood method in Mplus 8.4.

## 3 Results

### 3.1 Preliminary analyses

Descriptive statistics of main study variables are presented in Table 1. 27.5% (*n* = 75) of participants reported a history of CSA. Bivariate Pearson correlations and point-biserial correlations were used to assess the relationship between the variables of interest, namely CSA, RF, posttraumatic stress, sexual concerns, and borderline personality features (see Table 2). The results showed correlations of moderate strength between posttraumatic stress, sexual concerns, and borderline personality features. CSA had significant point-biserial correlations with posttraumatic stress and sexual concerns, while RF was correlated with posttraumatic stress, sexual concerns, and borderline personality features. The point-biserial correlation between CSA and RF did not reach significance in our sample (*r* = −0.07).

**Table 1:** Descriptive statistics for main study variables (*N* = 273).

Study variable	*n*	%	Mean (*SD*)	Minimum	Maximum
*Demographics*					
Age			15.5 (1.4)	12	17
Male	95	34.8%			
Female	178	65.2%			
Asian	9	3.3%			
Black or African-American	7	2.6%			
Hispanic or Latino	13	4.8%			
White/Caucasian	222	81.3%			
Multiracial or other	15	5.5%			
*RF and CSA*					
RF			8.7 (0.8)	4	10
CSA	75	27.5%			
*Male*	11	14.7%			
*Female*	64	85.3%			
*Trauma-related symptoms*					
Posttraumatic stress	53.7 (10.8)	35	84		
Sexual concerns	60.1 (21.9)	36	152		
Borderline personality features	70.9 (15.9)	32	112		

**Table 2:** Pearson correlations between CSA, RF, posttraumatic stress, sexual concerns, and borderline personality features.

	1	2	3	4	5	6	7
1. Age	–						
2. Gender	0.83	–					
3. CSA	0.00	−0.26^**^	–				
4. RF	0.17^**^	0.02	−0.07	–			
5. PTS	−0.13^*^	−0.04	0.20^**^	−0.32^**^	–		
6. SC	−0.09	−0.20^**^	0.22^**^	−0.29^**^	0.44^**^	–	
7. BPFS	−0.12	−0.22^**^	0.10	−0.34^**^	0.47^**^	0.50^**^	–

CSA, childhood sexual abuse; RF, reflective functioning; PTS, posttraumatic stress; SC, sexual concerns; BPFS, borderline personality features; gender: 0 = female, 1 = male. ^*^p < 0.05, ^**^p < 0.01.

### 3.2 Structural equation modeling (SEM)

**Figure 1:**
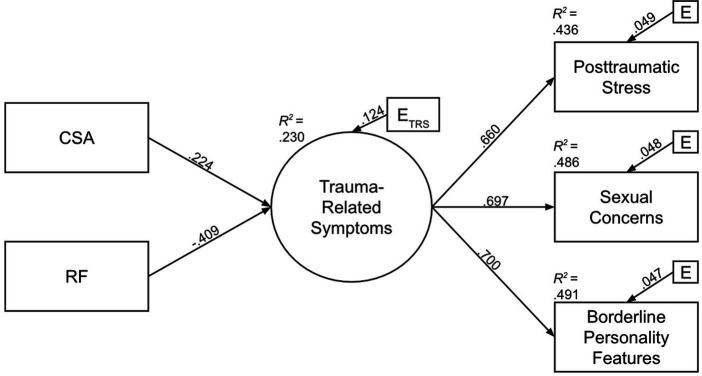
Structural equation model: Relative contributions of CSA and RF to trauma-related symptoms (posttraumatic stress, sexual concerns, and borderline personality features). Coefficients are standardized (β); all associations are significant using a *p* = 0.01 threshold.

The full results of the SEM are available in Figure 1 (*N* = 273). According to guidelines, the model showed an excellent fit with a non-significant χ2 test (*p* = 0.118), a RMSEA of 0.056, a SRMR of 0.028, a CFI of 0.984 and a TLI of 0.965. First, regarding the measurement model, the latent variable (trauma-related symptoms) explained 43.3% of the variance of posttraumatic stress, 48.6% of the variance of sexual concerns, and 49.1% of the variance of borderline personality features. The structural model aimed at examining the relative contribution of CSA and RF to trauma-related symptoms. Both CSA (*β* = 0.224, *p* = 0.001) and RF (*β* = −0.409, *p* < 0.001) were significantly associated with trauma-related symptoms. We then examined the unique contributions of the independent variables to determine the variance of the trauma-related symptoms they explained. CSA explained 5.0% of the variance of trauma-related symptoms and RF explained 16.7% of trauma-related symptoms. In total, when we consider both the unique and the shared contribution of the independent variables, the model explained 23.0% of the variance of trauma-related symptoms.

## 4 Discussion

The aim of this study was to examine the relationships between mentalizing, CSA, and trauma-related symptoms in a sample of adolescent psychiatric inpatients. Our main objective was to test a model where both CSA and mentalizing would have direct effects on trauma-related symptoms including posttraumatic stress, borderline personality features, and sexual concerns. The prevalence of CSA in this adolescent psychiatric inpatient sample was 27.5%, in line with previous studies such as that of Robin et al. (2023) who reported a prevalence range of 28.5%. In terms of our principal objective, the findings provide support for our hypothesized model where both CSA and mentalizing have direct effects on a latent factor constituted of trauma-related symptoms including posttraumatic stress, borderline personality features, and sexual concerns. Furthermore, and contrary to expectation, the direct effect of mentalizing on trauma-related symptoms was stronger than the direct effect of CSA on trauma-related symptoms. In fact, mentalizing explained 3 times more variance of the trauma-related symptoms than CSA. Furthermore, CSA and mentalizing made independent contributions to explaining variance in trauma-related symptoms.

As hypothesized, CSA had a positive direct effect on trauma-related symptoms, but the effect was weak. Regarding the specific components of trauma-related symptoms, CSA being associated with increased posttraumatic stress in adolescents is in line with previous research (Trickett et al., 2011; Alink et al., 2012; Nooner et al., 2012; Pérez-Fuentes et al., 2013; Hébert et al., 2014; Panagioti et al., 2015; Daigneault et al., 2016; Ford and Courtois, 2021), and extends knowledge by showing that this is also the case in a clinical sample of adolescents. In addition to posttraumatic stress, CSA was also associated with increased sexual concerns, in line with previous research reporting this association (Briere and Elliott, 2003; Ensink et al., 2018; Abu-Raya and Gewirtz-Meydan, 2023; Gewirtz-Meydan and Lassri, 2023) including in adolescents (Fergusson et al., 1997; Daigneault et al., 2016; Gewirtz-Meydan and Godbout, 2023). In terms of clinical implications, this suggests that CSA-related sexual concerns should be assessed alongside other trauma-related sequelae.

Regarding mentalizing and trauma-related symptoms, as hypothesized, better mentalizing had a negative direct effect on trauma-related symptoms. This finding is consistent with previous research showing that mentalizing deficits are associated with trauma-related psychopathology (Sharp et al., 2011, 2013; Chiesa and Fonagy, 2014; Sharp and Vanwoerden, 2015; Ensink et al., 2017a,b,c,d, 2023; Duval et al., 2018; Huang et al., 2020; Doba et al., 2022; Martin-Gagnon et al., 2023). More specifically, better mentalizing was associated with fewer posttraumatic stress symptoms, sexual concerns, and borderline personality features. These findings are in line with that of previous studies in which impaired mentalizing was associated with greater symptoms of BPD (Sharp et al., 2011, 2013; Sharp and Vanwoerden, 2015; Duval et al., 2018; Martin-Gagnon et al., 2023) and PTSD (Huang et al., 2020; Doba et al., 2022; Ensink et al., 2023). To the best of our knowledge, the present study is the first to demonstrate that better mentalizing is associated with fewer sexual concerns. This extends previous research that showed that in children, CSA was sequentially associated through mentalizing and dissociation with sexualization (Ensink et al., 2017a,b,c,d). It is possible, as theorized by Bigras (Bigras et al., 2020), that better mentalizing promotes a sense of positive identity and sense of self, which may be associated with reduced sexual concerns.

A key finding of this study is that the direct effects between mentalizing and trauma-related symptoms were unexpectedly stronger than between CSA and trauma-related symptoms. While the findings are broadly in line with mentalizing being a resilience factor in youth (Ensink et al., 2016, 2021; Borelli et al., 2018), it extends previous research by demonstrating this relation in adolescent psychiatric inpatients. Additionally, mentalizing explained more variance in a factor constituted of trauma symptoms. The latent trauma factor was good at explaining all of the trauma-related study variables (borderline personality features, posttraumatic stress, and sexual concerns) with a high shared variance. This suggests that despite CSA and mentalizing having independent direct effects on trauma-related symptoms, these three variables are closely intertwined and merit further study.

The present model of independent direct effects from CSA and mentalizing to trauma-related symptoms in adolescent psychiatric inpatients diverges from the mediational model found in child CSA survivors (Ensink et al., 2016). In adolescence, mentalizing may become relatively more independent from CSA, possibly because adolescents gain access to new opportunities and relationships in which they can develop mentalizing. Alternatively, when CSA occurs during adolescence, mentalizing abilities may already be relatively established. It is also possible that the severity of CSA may be somewhat lower in psychiatric inpatients compared to survivors seeking psychological help for CSA-related impacts. However, to our minds, the model identified in the present study has particular clinical utility and is in line with the trend to provide trauma-informed treatment where trauma is more directly addressed. If CSA has direct effects on trauma-related symptoms, as we have shown, by implication this suggests that interventions should also address CSA and the processes through which CSA impacts symptomatology. Rather than mediation, the findings of our study suggest that it is equally important to consider independent paths from CSA and mentalizing to trauma-related symptoms.

### 4.1 Study strengths and limitations

The study has a number of strengths including the relatively large sample size and the use of a difficult to recruit population of adolescent psychiatric inpatients. Furthermore, three measures were used to determine CSA, including two interviews and one self-report questionnaire. However, the study also has important limitations that need to be considered. First, the cross-sectional nature of this study limits the extent to which causality can be assumed. Also, the sample was recruited at a private inpatient facility in the United States and was primarily Caucasian. As such, the findings may not be generalizable to adolescent inpatients from different racial, ethnic, and socio-economic groups. Additionally, retrospective self-reports of maltreatment were used instead of forensic records. Another limitation concerns the treatment of CSA as a unitary phenomenon, although it covers a heterogeneous spectrum of experiences. We did not obtain information regarding the type, duration, frequency, or chronicity of abuse, the relationship with the abuser, the age at which the abuse occurred, whether the youth disclosed the abuse, and whether they were believed, supported, and protected. As a result, it was not possible to conduct analyses comparing intra-familial CSA with extra-familial CSA, or to examine whether more severe maltreatment, such as chronic penetrative CSA, was associated with worse outcomes. As a result, the findings should be seen as being representative of CSA in adolescent psychiatric inpatients and not in CSA survivors who, for example, have CSA-related forensic records or were involved with child protection services. In regard to the latter group, future studies might consider comparing these CSA survivors with a control group. Furthermore, we did not consider the presence and influence of other types of maltreatment, although CSA is known to occur together with other types of abuse and neglect (Finkelhor et al., 2007). As such, the present study does not enable us to disentangle the unique contribution of CSA or to pinpoint to what extent impacts are related to CSA in combination with other forms of maltreatment. Lastly, mentalizing was assessed using only one measure, despite the fact that it is a multidimensional ability. It is thus possible that CSA-associated impacts could become evident when other mentalizing measures are used.

### 4.2 Clinical implications

Taken together, our findings have important clinical implications and suggest that mentalizing is an important resilience process associated with fewer trauma-related symptoms. We take the finding that mentalizing had a stronger direct effect and explained more variance in trauma-related symptoms to mean that interventions aimed at enhancing mentalizing and therapies like Mentalization Based Treatment (MBT) should be effective in reducing trauma-related symptoms. However, given that most Mentalization Based Treatments are designed to enhance mentalizing and reduce BPD-associated prementalizing, they may need to be adapted to more optimally address CSA-related trauma impacts. Based on our previous work regarding the importance of mentalizing trauma and trauma-related impacts (Ensink et al., 2014; Berthelot et al., 2015, 2022), therapeutic interventions that can support mentalizing about trauma and its impacts (for example: on personality, trust, emotional and interpersonal reactions, sexuality and revictimization) could facilitate recovery for CSA survivors and reduce the risk of revictimization.

### 4.3 Conclusion

The findings enhance our understanding of the associations between CSA, mentalizing, and trauma-related symptoms in adolescent psychiatric inpatients. Both CSA and mentalizing had independent direct effects on trauma-related symptoms. Unexpectedly, the effect of mentalizing was stronger than that of CSA, with mentalizing explaining 16.7% of variance in trauma-related symptoms and CSA explaining 5%. Consistent with the notion that mentalizing is a resilience factor in the context of CSA, better mentalizing was associated with fewer borderline personality features, reduced posttraumatic stress, and fewer sexual concerns. Therapies that are focused on developing mentalizing, such as MBT, while also being trauma-informed may palliate trauma-related symptoms.

## Data availability statement

The data analyzed in this study is subject to the following licenses/restrictions: Anonymized data. Requests to access these datasets should be directed to csharp2@central.uh.edu.

## Ethics statement

The studies involving humans were approved by 1. University of Houston Committee for the Protection of Human Subjects 2. Baylor College of Medicine IRB. The studies were conducted in accordance with the local legislation and institutional requirements. Written informed consent for participation in this study was provided by the participants’ legal guardians/next of kin.

## Author contributions

MW: Conceptualization, Writing – original draft, Writing – review & editing, Formal analysis. MB: Conceptualization, Formal analysis, Writing – original draft, Writing - review & editing. CS: Data curation, Methodology, Project administration, Funding acquisition, Writing – review & editing. KE: Conceptualization, Supervision, Writing – review & editing, Formal analysis.
